# Parliament and the Profession

**Published:** 1916-04-22

**Authors:** 


### PARLIAMENT AND THE PROFESSION.

### Reduction in Medical Fees.

In the Committee stage of the Local Government (Emergency Provisions) Bill, when Clause 5 of Part II., which provides that the fee paid to a medical practitioner for the notification of infectious diseases should be reduced from 2s. 6d. to 1s. (The Hospital, April 8, p. 37), was reached a strong protest was made against this reduction by Sir Philip Magnus. He proposed that for the notification of infectious diseases should be case of measles, in which, "on account of the greater ease with which it is diagnosed," the fee should be 1s. Sir Philip objected to the statement of the Retrenchment Committee, that "after all almost the whole work is clerical labour." Mr. J. Samuel, in supporting the amendment, instanced a case in one town where the want of proper notification of an outbreak of smallpox cost the local authority £35,000 to stamp out, and urged that the proposal to reduce the fee might have a tendency to produce very great discouragement among the doctors. Mr. Boyton also protested against the reduction. Mr. Hayes Fisher, who is in charge of the Bill, could not hold out any hope that the President of the Local Government Board would forego this clause. He had received, he said, a deputation from the British Medical Association on the matter, and they themselves stated that the 1s. would be sufficient for notifying measles. But the medical advisers of the Board could not differentiate between different diseases, and considered that if 1s. was sufficient for the notification of measles, then 1s. was sufficient for drawing up a simple form in regard to other diseases notified. The amendment was negatived.

### Local Authorities and Venereal Diseases.

The motion of Mr. King, that such provisions should be inserted in the Local Government (Emergency Provisions) Bill as may be necessary to carry into effect the recommendations contained in the Report of the Royal Commission on Venereal Diseases so far as these recommendations relate to the Local Government Board or to local authorities, was not allowed by the Speaker.

### War-Time Work of Lunacy Commissioners.

The Home Secretary, in a statement with regard to the functions of the two Lunacy Commissioners who have been temporarily lent to the War Office, said that they were both medical men with high qualifications and wide experience in hospital organisation and administration. It was mainly through their advice and action that the Board of Control had been able to place at the disposal of the War Office nearly 20,000 well-equipped beds for sick and wounded soldiers. They are not receiving any payment from the War Office. The number of hospitals so far provided by the Board of Control is thirteen, and another will be ready shortly. In reply to another question, it was stated that the Lunacy Commissioner who has been absent on military duty since the beginning of the war is Major B. T. Hodgson. His duties, said Mr. Tennant, are in no way associated with his civil office and, in accordance with the general practice of the Civil Service, he is receiving his civil salary minus his military pay.

### Other Matters of Interest.

Expenses of the Royal Commission on Venereal Diseases.—The expenses of this Commission, stated Mr. McKenna, amounted to £3,585, including £980 for reports, appendices, and index.

British Invalid Prisoners in Switzerland.—Replying to Mr. Malcolm, Mr. Tennant admitted that the arrangements for transferring British invalid prisoners of war to sanatoria in Switzerland had not yet been completed.

Treatment of the Wounded.—The War Office, said Mr. Tennant in answer to a question, is not in possession of statistics showing the number of cases of compound fracture in which, through the occurrence of sepsis, leg or limb has been sacrificed.

Tuberculous Soldiers.—In future the work of assisting the treatment of tuberculous soldiers will be entrusted to the Statutory Committee on Soldiers' and Sailors' Pensions. This was the effect of a statement made by Mr. Montagu in reply to Sir Henry Craik.

Medical Men Over Military Age.—Replying to Sir J. Lonsdale, Mr. Tennant said the War Office was endeavouring to carry out the policy that medical men of more advanced years rendered the country the better service by undertaking the care of the civil population and thus setting free those of military age who are physically fit for duty at home or abroad.

Medical Boards.—In the course of his reply to a series of questions respecting the medical examination of recruits in the Isle of Wight, Mr. Tennant stated that owing to the limited number of medical officers available it would not be possible to establish medical boards at places other than area headquarters.

Verandah Huts.—A special type of hut has been designed with verandahs for the treatment of some military cases though not necessarily for consumptive cases, but Mr. Tennant stated, in answer to a question, that he had no data as to the number of patients who could be accommodated in existing verandahs.

Special Work in the R.A.M.C.—Mr. R. McNeill again returned to the question of utilising the services of medical men for such duties as their special acquirements best fit them. Mr. Tennant said that this was the policy adopted as far as possible, but it was impossible to allow each individual to select the work which he considered himself most competent to undertake.

Soldiers Treated in Hospitals.—Replying to Major Astor, the Under-Secretary for War stated that hospital treatment is continued until a cure is effected or until it is obvious that the soldier will not be medically fit to remain in the Service. In no circumstances is a soldier discharged from hospital (except to special institutions) unless he is able to leave without detriment to his health.

Infant Welfare.—The President of the Local Government Board cannot undertake to issue instructions to local authorities that an expenditure not exceeding one halfpenny in the pound may be judiciously undertaken on promoting infant welfare, but in answer to a supplementary question by Mr. King he said the Board had suggested to medical officers of health that health visitors should be employed in the proportion of not less than two for every 1,000 births.

